# Tricarbonyl[tris(1-methyl-1*H*-imidazol-2-yl-κ*N* ^3^)methanol]manganese(I) trifluoromethanesulfonate

**DOI:** 10.1107/S1600536812035891

**Published:** 2012-08-23

**Authors:** Guido J. Reiss, Peter C. Kunz

**Affiliations:** a Institut für Anorganische Chemie und Strukturchemie, Lehrstuhl II: Material- und Strukturforschung, Heinrich-Heine-Universität Düsseldorf, Universitätsstrasse 1, D-40225 Düsseldorf, Germany; b Institut für Pharmazeutische Chemie, Heinrich-Heine-Universität Düsseldorf, Universitätsstrasse 1, D-40225 Düsseldorf, Germany

## Abstract

In the title compound, [Mn(C_13_H_16_N_6_O)(CO)_3_](CF_3_O_3_S), the Mn^I^ atom has a slightly distorted octa­hedral geometry. The three CO ligands have C—Mn—C angles in the range 89.44 (10)–92.31 (9)°, while the three N atoms of the tripodal ligand form significantly smaller N—Mn—N angles of 82.76 (2)–85.51 (6)°. The three N atoms of the tripodal ligand and the three carbonyl ligands coordinate facially. In the crystal, the trifluoro­methane­sulfonate counter anion is connected by a medium-strength O—H⋯O hydrogen bond to the hydroxyl group of the manganese complex.

## Related literature

For the structures of related complexes, see: Niesel *et al.* (2008 ▶); Herrick *et al.* (2008 ▶); Kunz *et al.* (2009 ▶). For details of the chemistry of tris­(imidazolyl-2-yl)carbinol ligands, see: Stamatatos *et al.* (2009 ▶); Breslow *et al.* (1983 ▶); Tang *et al.* (1978 ▶). For details of the chemistry of Mn(CO)_3_ complexes, see: Kreiter *et al.* (1994 ▶, 1995 ▶); Brückmann *et al.* (2011 ▶); Huber *et al.* (2012 ▶); Berends & Kurz (2012 ▶).
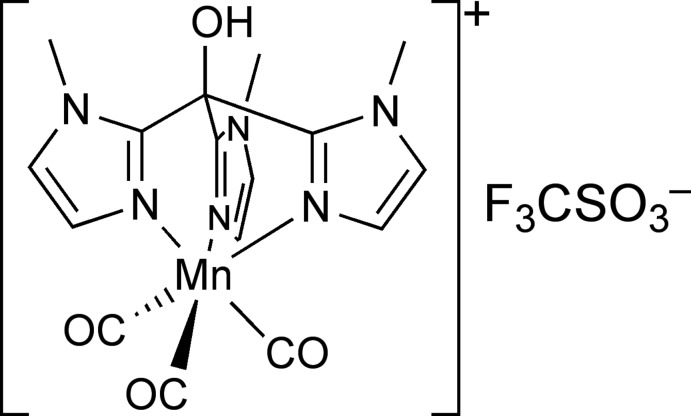


## Experimental

### 

#### Crystal data

[Mn(CO)_3_(C_13_H_16_N_6_O)](CF_3_O_3_S)
*M* *_r_* = 560.36
Monoclinic, 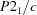

*a* = 12.16673 (18) Å
*b* = 15.5692 (2) Å
*c* = 12.6240 (2) Å
β = 104.6721 (16)°
*V* = 2313.33 (6) Å^3^
*Z* = 4
Mo *K*α radiation
μ = 0.74 mm^−1^
*T* = 290 K
0.80 × 0.74 × 0.40 mm

#### Data collection

Oxford Xcalibur diffractometer with Eos detector
Absorption correction: multi-scan *CrysAlis PRO* (Oxford Diffraction, 2009 ▶) *T*_min_ = 0.805, *T*_max_ = 1.000
95191 measured reflections
6746 independent reflections
5888 reflections with *I* > 2σ(*I*)
*R*_int_ = 0.027

#### Refinement

*R*[*F* ^2^ > 2σ(*F* ^2^)] = 0.040
*wR*(*F* ^2^) = 0.084
*S* = 1.06
6746 reflections
330 parameters
H atoms treated by a mixture of independent and constrained refinement
Δρ_max_ = 0.37 e Å^−3^
Δρ_min_ = −0.51 e Å^−3^

### 

Data collection: *CrysAlis PRO* (Oxford Diffraction, 2009 ▶); cell refinement: *CrysAlis PRO*; data reduction: *CrysAlis PRO*; program(s) used to solve structure: *SHELXS97* (Sheldrick, 2008 ▶); program(s) used to refine structure: *SHELXL97* (Sheldrick, 2008 ▶); molecular graphics: *DIAMOND* (Brandenburg, 2011 ▶); software used to prepare material for publication: *publCIF* (Westrip, 2010 ▶).

## Supplementary Material

Crystal structure: contains datablock(s) I, global. DOI: 10.1107/S1600536812035891/pk2428sup1.cif

Structure factors: contains datablock(s) I. DOI: 10.1107/S1600536812035891/pk2428Isup2.hkl

Additional supplementary materials:  crystallographic information; 3D view; checkCIF report

## Figures and Tables

**Table 1:** Hydrogen-bond geometry (Å, °)

*D*—H⋯*A*	*D*—H	H⋯*A*	*D*⋯*A*	*D*—H⋯*A*
O1—H1⋯O5	0.72 (2)	1.98 (2)	2.694 (2)	175 (2)

**Fig. 1.**
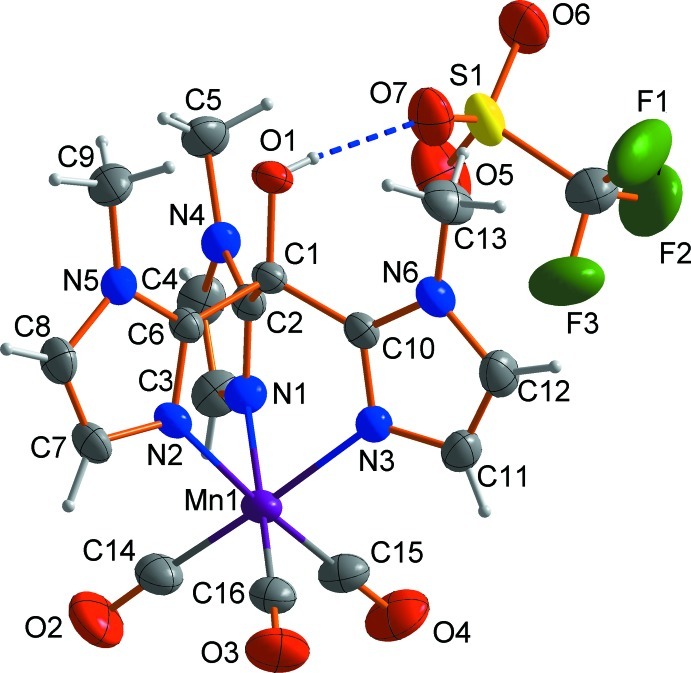
The asymmetric unit of the title compound.

## supplementary crystallographic information

### Comment

The chemistry of manganese carbonyl complexes is of significant interest for at least two reasons. On the one hand there is a long standing interest in simple organometallic Mn(CO)_3_ complexes, reflected by more than 2200 structures reported in the CCDC. On the other hand they are known to undergo a plethora of photochemical reactions, *e.g.* photochemical mediated cycloaddition reactions yielding complex organic ligand systems coordinated to a manganese center (*e.g.* Kreiter *et al.*, 1994, 1995). Recently, manganese tricarbonyl complexes of tripodal *N*,*N*,*N*-ligands, like tris(imidazolyl)carbinols (Breslow *et al.*, 1983; Tang *et al.* 1978), have been shown to be photoinduced CO-releasing molecules (photoCORMs). The CO-release characteristics, *e.g.* the rate and half-life time for the release, are dependent on the ligands used to stabilize the Mn(CO)_3_ core (Huber *et al.*, 2012; Berends & Kurz, 2012; Brückmann *et al.*, 2011; Kunz *et al.*, 2009; Niesel *et al.*, 2008). The manganese complex cation shows *N*,*N*,*N*-coordination in the solid state, which has also been observed for the corresponding rhenium(I) complex, in which the carbinol OH has been methylated (Herrick *et al.*, 2008). The spectroscopic data in solution (IR and NMR, Huber *et al.*, 2012) of the title compound are in accord with *C*_3_*_v_* symmetry and therefore with the *N*,*N*,*N*-coordination found in the solid state. This indicates that coordination of the carbinol OH group is not favored, as found in other carbinol ligands (Stamatatos *et al.*, 2009; Herrick *et al.*, 2008).

The asymmetric unit of the title structure, consisting of a complex manganese cation and the trifluoromethanesulfonate counteranion, is shown in Fig. 1. The coordination polyhedron around the central manganese(I) atom is slightly distorted from octahedral symmetry. All Mn—N and Mn—C distances are in the expected range for a manganese(I) tricarbonyl complex. The three angles between the three CO ligands are near 90°, which is typical for the Mn(CO)_3_ fragment (*e.g.* Kreiter *et al.*, 1995). The three angles N—Mn—N are significantly smaller than 90° (82.76 (2) to 85.51 (6)°), which is a result of the bite angle the tripodal ligand. The complex cation is connected to the trifluoromethanesulfonate counter-anion by only one O—H···O hydrogen bond, between the carbinol group of the complex cation and one of the O atoms of the trifluoromethanesulfonate anion.

### Experimental

The synthesis of the title compound was performed as recently reported (Huber *et al.* 2012). The title compound was crystallized from methanol solution by slow vapor diffusion of diethyl ether to yield yellow crystals. ^1^H NMR (200 MHz, [D_4_]methanol): δ = 4.12 (s, 9 H, NCH_3_), 7.17 (d, ^3^J_H,H_ = 1.4 Hz, 3 H, H_im_), 7.42 (d, ^3^J_H,H_ = 1.4 Hz, 3 H, H_im_) p.p.m. ^13^C{^1^H} NMR (125 MHz, [D_4_]methanol): δ = 37.0, 78.4, 126.1, 132.0, 145.1 p.p.m. ESI-MS (MeOH): m/*z* (%) = 411.1 (36) [*M*]^+^, 354.9 (12) [*M*-2CO]^+^, 327.3 (100) [*M*-3CO]^+^. C_17_H_16_F_3_MnN_6_O_7_S (560.3): calcd. C 36.44, H 2.88, N 15.00; found C 36.75, H 2.55, N 14.86. IR (KBr): ν = 2044, 1936, 1907 cm^-1^. IR (CH_2_Cl_2_): ν = 2037, 1935 cm^-1^.

### Refinement

All H-atom positions were identified in difference Fourier maps. In the later stages of refinement the H atoms of the methyl groups and the H atoms of the rings of the tripodal ligand were refined using a riding model. The *U*_iso_ values of the methyl H atoms were set to 1.5 times the equivalent isotropic displacement parameter of the C atom they are attached to. The *U*_iso_ values of the H atoms at the rings of the tripodal ligand were refined freely. The coordinates and the *U*_iso_ value of the H atom of the carbinol function were refined freely.

### Crystal data

**Table:**

[Mn(C_13_H_16_N_6_O)(CO)_3_]·CF_3_O_3_S	*F*(000) = 1136
*M**_r_* = 560.36	*D*_x_ = 1.609 Mg m^−^^3^
Monoclinic, *P*2_1_/*c*	Mo *K*α radiation, λ = 0.71073 Å
Hall symbol: -P 2ybc	Cell parameters from 50962 reflections
*a* = 12.16673 (18) Å	θ = 3.0–31.7°
*b* = 15.5692 (2) Å	µ = 0.74 mm^−^^1^
*c* = 12.6240 (2) Å	*T* = 290 K
β = 104.6721 (16)°	Block, yellow
*V* = 2313.33 (6) Å^3^	0.80 × 0.74 × 0.40 mm
*Z* = 4	

### Data collection

**Table:**

Oxford Xcalibur with Eos detector? diffractometer	6746 independent reflections
Radiation source: fine-focus sealed tube	5888 reflections with *I* > 2σ(*I*)
Graphite monochromator	*R*_int_ = 0.027
Detector resolution: 16.2711 pixels mm^-1^	θ_max_ = 30.0°, θ_min_ = 3.0°
ω scans	*h* = −17→17
Absorption correction: multi-scan *CrysAlis PRO* (Oxford Diffraction, 2009)	*k* = −21→21
*T*_min_ = 0.805, *T*_max_ = 1.000	*l* = −17→17
95191 measured reflections	

### Refinement

**Table:**

Refinement on *F*^2^	Secondary atom site location: difference Fourier map
Least-squares matrix: full	Hydrogen site location: inferred from neighbouring sites
*R*[*F*^2^ > 2σ(*F*^2^)] = 0.040	H atoms treated by a mixture of independent and constrained refinement
*wR*(*F*^2^) = 0.084	*w* = 1/[σ^2^(*F*_o_^2^) + (0.015*P*)^2^ + 2.*P*] where *P* = (*F*_o_^2^ + 2*F*_c_^2^)/3
*S* = 1.06	(Δ/σ)_max_ = 0.001
6746 reflections	Δρ_max_ = 0.37 e Å^−^^3^
330 parameters	Δρ_min_ = −0.51 e Å^−^^3^
0 restraints	Extinction correction: *SHELXL97* (Sheldrick, 2008), Fc^*^=kFc[1+0.001xFc^2^λ^3^/sin(2θ)]^-1/4^
Primary atom site location: structure-invariant direct methods	Extinction coefficient: 0.00166 (17)

### Special details

**Table:**

Geometry. All e.s.d.'s (except the e.s.d. in the dihedral angle between two l.s. planes) are estimated using the full covariance matrix. The cell e.s.d.'s are taken into account individually in the estimation of e.s.d.'s in distances, angles and torsion angles; correlations between e.s.d.'s in cell parameters are only used when they are defined by crystal symmetry. An approximate (isotropic) treatment of cell e.s.d.'s is used for estimating e.s.d.'s involving l.s. planes.
Refinement. Refinement of *F*^2^ against ALL reflections. The weighted *R*-factor *wR* and goodness of fit *S* are based on *F*^2^, conventional *R*-factors *R* are based on *F*, with *F* set to zero for negative *F*^2^. The threshold expression of *F*^2^ > σ(*F*^2^) is used only for calculating *R*-factors(gt) *etc*. and is not relevant to the choice of reflections for refinement. *R*-factors based on *F*^2^ are statistically about twice as large as those based on *F*, and *R*-factors based on ALL data will be even larger.

### Fractional atomic coordinates and isotropic or equivalent isotropic displacement parameters (Å^2^)

**Table:**

	*x*	*y*	*z*	*U*_iso_*/*U*_eq_	
Mn1	0.10254 (2)	0.295254 (17)	0.16048 (2)	0.03656 (8)	
O1	0.35083 (11)	0.49536 (9)	0.35279 (11)	0.0412 (3)	
H1	0.4054 (19)	0.4867 (14)	0.3424 (18)	0.044 (6)*	
N1	0.19095 (13)	0.38711 (10)	0.10358 (12)	0.0389 (3)	
C1	0.27555 (12)	0.43432 (10)	0.29428 (13)	0.0315 (3)	
O2	−0.11219 (16)	0.32763 (15)	−0.00447 (16)	0.0896 (6)	
N2	0.07555 (11)	0.39388 (10)	0.26080 (12)	0.0354 (3)	
C2	0.25808 (13)	0.44160 (10)	0.17035 (14)	0.0333 (3)	
O3	−0.02144 (16)	0.17630 (12)	0.27000 (15)	0.0730 (5)	
N3	0.25271 (12)	0.27691 (9)	0.27527 (12)	0.0370 (3)	
C3	0.18358 (19)	0.41522 (14)	−0.00092 (16)	0.0503 (5)	
H3	0.1420	0.3890	−0.0647	0.060 (7)*	
O4	0.16020 (19)	0.15860 (12)	0.02292 (16)	0.0834 (6)	
N4	0.29270 (13)	0.50443 (10)	0.11247 (13)	0.0416 (3)	
C4	0.2463 (2)	0.48683 (15)	0.00382 (17)	0.0542 (5)	
H4	0.2563	0.5185	−0.0555	0.070 (8)*	
N5	0.11867 (12)	0.50560 (9)	0.37074 (12)	0.0369 (3)	
C5	0.35957 (19)	0.58200 (14)	0.1479 (2)	0.0597 (6)	
H5A	0.3280	0.6129	0.1991	0.090*	
H5B	0.3579	0.6177	0.0855	0.090*	
H5C	0.4367	0.5664	0.1823	0.090*	
N6	0.38841 (12)	0.31476 (10)	0.41865 (13)	0.0418 (3)	
C6	0.15755 (13)	0.44636 (10)	0.31180 (13)	0.0314 (3)	
C7	−0.02132 (14)	0.42127 (13)	0.28780 (16)	0.0434 (4)	
H7	−0.0929	0.3967	0.2634	0.051 (6)*	
C8	0.00426 (15)	0.48947 (13)	0.35522 (16)	0.0452 (4)	
H8	−0.0459	0.5200	0.3857	0.052 (6)*	
C9	0.18119 (19)	0.57170 (14)	0.44384 (18)	0.0539 (5)	
H9A	0.2387	0.5451	0.5008	0.081*	
H9B	0.1297	0.6031	0.4758	0.081*	
H9C	0.2163	0.6103	0.4030	0.081*	
C10	0.30911 (13)	0.34236 (11)	0.32977 (13)	0.0332 (3)	
C11	0.30031 (17)	0.20377 (13)	0.32892 (18)	0.0476 (4)	
H11	0.2788	0.1478	0.3077	0.051 (6)*	
C12	0.38308 (18)	0.22648 (14)	0.41719 (18)	0.0522 (5)	
H12	0.4282	0.1894	0.4678	0.064 (7)*	
C13	0.46166 (19)	0.36356 (16)	0.50876 (17)	0.0602 (6)	
H13A	0.5207	0.3913	0.4834	0.090*	
H13B	0.4950	0.3252	0.5677	0.090*	
H13C	0.4172	0.4061	0.5342	0.090*	
C14	−0.02874 (18)	0.31700 (15)	0.05903 (18)	0.0533 (5)	
C15	0.13726 (19)	0.21210 (14)	0.07542 (17)	0.0511 (5)	
C16	0.02768 (17)	0.22112 (13)	0.22707 (17)	0.0473 (4)	
S1	0.61262 (4)	0.42786 (4)	0.23478 (5)	0.05368 (14)	
F1	0.7020 (2)	0.30397 (12)	0.36557 (18)	0.1337 (10)	
F2	0.7028 (2)	0.28311 (15)	0.2001 (2)	0.1324 (9)	
F3	0.55074 (19)	0.26955 (13)	0.24881 (19)	0.1151 (7)	
O5	0.55176 (13)	0.45179 (12)	0.31485 (14)	0.0660 (4)	
O6	0.72136 (14)	0.46749 (12)	0.25442 (18)	0.0790 (6)	
O7	0.54709 (19)	0.42639 (18)	0.12357 (16)	0.1040 (8)	
C17	0.6452 (2)	0.31564 (18)	0.2657 (3)	0.0738 (7)	

### Atomic displacement parameters (Å^2^)

**Table:**

	*U*^11^	*U*^22^	*U*^33^	*U*^12^	*U*^13^	*U*^23^
Mn1	0.03490 (13)	0.03877 (14)	0.03562 (13)	−0.00670 (10)	0.00821 (10)	−0.00727 (10)
O1	0.0303 (6)	0.0422 (7)	0.0499 (7)	−0.0083 (5)	0.0082 (5)	−0.0103 (6)
N1	0.0442 (8)	0.0417 (8)	0.0332 (7)	−0.0027 (6)	0.0141 (6)	−0.0036 (6)
C1	0.0252 (7)	0.0340 (8)	0.0351 (8)	−0.0031 (6)	0.0074 (6)	−0.0034 (6)
O2	0.0612 (11)	0.1112 (17)	0.0761 (13)	0.0058 (11)	−0.0201 (9)	−0.0129 (12)
N2	0.0264 (6)	0.0435 (8)	0.0372 (7)	−0.0023 (5)	0.0095 (5)	−0.0053 (6)
C2	0.0310 (7)	0.0340 (8)	0.0373 (8)	0.0012 (6)	0.0131 (6)	0.0011 (6)
O3	0.0760 (11)	0.0740 (12)	0.0726 (11)	−0.0295 (9)	0.0254 (9)	0.0044 (9)
N3	0.0336 (7)	0.0352 (7)	0.0427 (8)	−0.0006 (5)	0.0105 (6)	0.0001 (6)
C3	0.0645 (13)	0.0548 (12)	0.0345 (9)	−0.0001 (10)	0.0178 (9)	−0.0020 (8)
O4	0.1109 (16)	0.0656 (11)	0.0833 (13)	−0.0103 (11)	0.0426 (12)	−0.0355 (10)
N4	0.0414 (8)	0.0384 (8)	0.0477 (8)	−0.0001 (6)	0.0160 (7)	0.0078 (6)
C4	0.0674 (13)	0.0569 (12)	0.0432 (10)	0.0030 (10)	0.0234 (10)	0.0109 (9)
N5	0.0364 (7)	0.0379 (7)	0.0371 (7)	0.0036 (6)	0.0104 (6)	−0.0036 (6)
C5	0.0559 (12)	0.0425 (11)	0.0765 (15)	−0.0102 (9)	0.0092 (11)	0.0172 (10)
N6	0.0339 (7)	0.0481 (9)	0.0410 (8)	−0.0008 (6)	0.0049 (6)	0.0074 (7)
C6	0.0279 (7)	0.0362 (8)	0.0304 (7)	0.0006 (6)	0.0081 (6)	−0.0021 (6)
C7	0.0271 (7)	0.0551 (11)	0.0496 (10)	0.0005 (7)	0.0130 (7)	−0.0011 (8)
C8	0.0361 (9)	0.0535 (11)	0.0503 (10)	0.0088 (8)	0.0190 (8)	−0.0022 (9)
C9	0.0560 (12)	0.0498 (11)	0.0540 (12)	0.0018 (9)	0.0103 (9)	−0.0200 (9)
C10	0.0265 (7)	0.0385 (8)	0.0351 (8)	−0.0007 (6)	0.0085 (6)	0.0012 (6)
C11	0.0474 (10)	0.0362 (9)	0.0606 (12)	0.0013 (8)	0.0165 (9)	0.0071 (8)
C12	0.0461 (10)	0.0480 (11)	0.0603 (12)	0.0050 (9)	0.0095 (9)	0.0173 (9)
C13	0.0546 (12)	0.0722 (15)	0.0428 (11)	−0.0089 (11)	−0.0080 (9)	0.0071 (10)
C14	0.0488 (11)	0.0585 (12)	0.0485 (11)	−0.0059 (9)	0.0046 (9)	−0.0109 (9)
C15	0.0586 (12)	0.0473 (11)	0.0498 (11)	−0.0125 (9)	0.0180 (9)	−0.0102 (9)
C16	0.0444 (10)	0.0491 (11)	0.0464 (10)	−0.0101 (8)	0.0080 (8)	−0.0088 (8)
S1	0.0363 (2)	0.0724 (4)	0.0550 (3)	0.0099 (2)	0.0165 (2)	0.0156 (3)
F1	0.190 (2)	0.0667 (11)	0.1016 (15)	0.0248 (13)	−0.0427 (15)	0.0090 (10)
F2	0.1315 (18)	0.1085 (16)	0.174 (2)	0.0228 (14)	0.0696 (17)	−0.0448 (16)
F3	0.1188 (16)	0.0877 (13)	0.1425 (18)	−0.0411 (12)	0.0400 (14)	−0.0251 (13)
O5	0.0524 (9)	0.0804 (12)	0.0741 (11)	0.0120 (8)	0.0325 (8)	0.0101 (9)
O6	0.0489 (9)	0.0765 (12)	0.1204 (16)	−0.0028 (8)	0.0379 (10)	0.0115 (11)
O7	0.0862 (14)	0.161 (2)	0.0569 (11)	0.0263 (15)	0.0037 (10)	0.0251 (13)
C17	0.0713 (16)	0.0631 (15)	0.0827 (19)	−0.0039 (13)	0.0116 (14)	−0.0165 (14)

### Geometric parameters (Å, º)

**Table:**

Mn1—C15	1.799 (2)	N5—C8	1.379 (2)
Mn1—C16	1.804 (2)	N5—C9	1.461 (2)
Mn1—C14	1.808 (2)	C5—H5A	0.9600
Mn1—N1	2.0273 (15)	C5—H5B	0.9600
Mn1—N3	2.0441 (15)	C5—H5C	0.9600
Mn1—N2	2.0688 (14)	N6—C10	1.351 (2)
O1—C1	1.3946 (19)	N6—C12	1.376 (3)
O1—H1	0.72 (2)	N6—C13	1.467 (3)
N1—C2	1.322 (2)	C7—C8	1.347 (3)
N1—C3	1.371 (2)	C7—H7	0.9300
C1—C6	1.519 (2)	C8—H8	0.9300
C1—C10	1.525 (2)	C9—H9A	0.9600
C1—C2	1.529 (2)	C9—H9B	0.9600
O2—C14	1.135 (3)	C9—H9C	0.9600
N2—C6	1.324 (2)	C11—C12	1.346 (3)
N2—C7	1.375 (2)	C11—H11	0.9300
C2—N4	1.351 (2)	C12—H12	0.9300
O3—C16	1.141 (2)	C13—H13A	0.9600
N3—C10	1.320 (2)	C13—H13B	0.9600
N3—C11	1.376 (2)	C13—H13C	0.9600
C3—C4	1.343 (3)	S1—O6	1.4234 (17)
C3—H3	0.9300	S1—O7	1.428 (2)
O4—C15	1.142 (3)	S1—O5	1.4449 (16)
N4—C4	1.372 (3)	S1—C17	1.812 (3)
N4—C5	1.462 (3)	F1—C17	1.288 (3)
C4—H4	0.9300	F2—C17	1.314 (3)
N5—C6	1.344 (2)	F3—C17	1.325 (3)
			
C15—Mn1—C16	92.31 (9)	H5A—C5—H5C	109.5
C15—Mn1—C14	90.62 (10)	H5B—C5—H5C	109.5
C16—Mn1—C14	89.44 (10)	C10—N6—C12	106.58 (16)
C15—Mn1—N1	94.03 (8)	C10—N6—C13	130.15 (17)
C16—Mn1—N1	172.75 (7)	C12—N6—C13	123.03 (17)
C14—Mn1—N1	93.99 (9)	N2—C6—N5	111.42 (14)
C15—Mn1—N3	91.13 (8)	N2—C6—C1	118.49 (14)
C16—Mn1—N3	92.66 (8)	N5—C6—C1	130.06 (14)
C14—Mn1—N3	177.21 (8)	C8—C7—N2	109.01 (16)
N1—Mn1—N3	83.72 (6)	C8—C7—H7	125.5
C15—Mn1—N2	175.58 (8)	N2—C7—H7	125.5
C16—Mn1—N2	90.71 (8)	C7—C8—N5	107.26 (15)
C14—Mn1—N2	92.63 (8)	C7—C8—H8	126.4
N1—Mn1—N2	82.76 (6)	N5—C8—H8	126.4
N3—Mn1—N2	85.51 (6)	N5—C9—H9A	109.5
C1—O1—H1	106.7 (18)	N5—C9—H9B	109.5
C2—N1—C3	106.73 (16)	H9A—C9—H9B	109.5
C2—N1—Mn1	121.61 (11)	N5—C9—H9C	109.5
C3—N1—Mn1	130.91 (14)	H9A—C9—H9C	109.5
O1—C1—C6	110.92 (13)	H9B—C9—H9C	109.5
O1—C1—C10	113.15 (13)	N3—C10—N6	110.77 (15)
C6—C1—C10	105.41 (13)	N3—C10—C1	120.42 (14)
O1—C1—C2	113.20 (13)	N6—C10—C1	128.64 (15)
C6—C1—C2	104.38 (12)	C12—C11—N3	108.87 (18)
C10—C1—C2	109.14 (13)	C12—C11—H11	125.6
C6—N2—C7	106.01 (14)	N3—C11—H11	125.6
C6—N2—Mn1	122.61 (11)	C11—C12—N6	107.24 (17)
C7—N2—Mn1	131.37 (12)	C11—C12—H12	126.4
N1—C2—N4	110.29 (15)	N6—C12—H12	126.4
N1—C2—C1	120.54 (14)	N6—C13—H13A	109.5
N4—C2—C1	128.73 (15)	N6—C13—H13B	109.5
C10—N3—C11	106.49 (15)	H13A—C13—H13B	109.5
C10—N3—Mn1	120.98 (12)	N6—C13—H13C	109.5
C11—N3—Mn1	130.84 (13)	H13A—C13—H13C	109.5
C4—C3—N1	108.91 (18)	H13B—C13—H13C	109.5
C4—C3—H3	125.5	O2—C14—Mn1	177.5 (2)
N1—C3—H3	125.5	O4—C15—Mn1	178.9 (2)
C2—N4—C4	106.87 (16)	O3—C16—Mn1	177.9 (2)
C2—N4—C5	131.21 (17)	O6—S1—O7	116.17 (14)
C4—N4—C5	121.81 (17)	O6—S1—O5	112.63 (12)
C3—C4—N4	107.19 (17)	O7—S1—O5	115.74 (12)
C3—C4—H4	126.4	O6—S1—C17	103.75 (12)
N4—C4—H4	126.4	O7—S1—C17	103.49 (15)
C6—N5—C8	106.29 (14)	O5—S1—C17	102.74 (13)
C6—N5—C9	129.29 (15)	F1—C17—F2	108.8 (3)
C8—N5—C9	124.30 (16)	F1—C17—F3	108.1 (3)
N4—C5—H5A	109.5	F2—C17—F3	105.6 (2)
N4—C5—H5B	109.5	F1—C17—S1	112.37 (19)
H5A—C5—H5B	109.5	F2—C17—S1	111.0 (2)
N4—C5—H5C	109.5	F3—C17—S1	110.8 (2)
			
C15—Mn1—N1—C2	−135.39 (15)	C7—N2—C6—C1	−177.73 (15)
C14—Mn1—N1—C2	133.71 (15)	Mn1—N2—C6—C1	1.0 (2)
N3—Mn1—N1—C2	−44.69 (14)	C8—N5—C6—N2	−0.3 (2)
N2—Mn1—N1—C2	41.56 (13)	C9—N5—C6—N2	175.87 (18)
C15—Mn1—N1—C3	55.91 (19)	C8—N5—C6—C1	177.59 (17)
C14—Mn1—N1—C3	−35.00 (19)	C9—N5—C6—C1	−6.2 (3)
N3—Mn1—N1—C3	146.60 (18)	O1—C1—C6—N2	179.32 (15)
N2—Mn1—N1—C3	−127.15 (18)	C10—C1—C6—N2	−57.86 (18)
C16—Mn1—N2—C6	132.48 (15)	C2—C1—C6—N2	57.08 (19)
C14—Mn1—N2—C6	−138.05 (15)	O1—C1—C6—N5	1.5 (2)
N1—Mn1—N2—C6	−44.36 (14)	C10—C1—C6—N5	124.35 (18)
N3—Mn1—N2—C6	39.86 (14)	C2—C1—C6—N5	−120.71 (18)
C16—Mn1—N2—C7	−49.16 (17)	C6—N2—C7—C8	−0.4 (2)
C14—Mn1—N2—C7	40.31 (18)	Mn1—N2—C7—C8	−178.98 (13)
N1—Mn1—N2—C7	134.01 (17)	N2—C7—C8—N5	0.2 (2)
N3—Mn1—N2—C7	−141.77 (17)	C6—N5—C8—C7	0.1 (2)
C3—N1—C2—N4	0.9 (2)	C9—N5—C8—C7	−176.38 (18)
Mn1—N1—C2—N4	−170.19 (11)	C11—N3—C10—N6	−2.23 (19)
C3—N1—C2—C1	173.97 (15)	Mn1—N3—C10—N6	164.50 (11)
Mn1—N1—C2—C1	2.9 (2)	C11—N3—C10—C1	−178.01 (15)
O1—C1—C2—N1	178.58 (15)	Mn1—N3—C10—C1	−11.3 (2)
C6—C1—C2—N1	−60.70 (19)	C12—N6—C10—N3	1.9 (2)
C10—C1—C2—N1	51.60 (19)	C13—N6—C10—N3	−172.51 (19)
O1—C1—C2—N4	−9.8 (2)	C12—N6—C10—C1	177.26 (17)
C6—C1—C2—N4	110.93 (18)	C13—N6—C10—C1	2.8 (3)
C10—C1—C2—N4	−136.77 (17)	O1—C1—C10—N3	−173.45 (14)
C15—Mn1—N3—C10	143.22 (14)	C6—C1—C10—N3	65.17 (18)
C16—Mn1—N3—C10	−124.41 (14)	C2—C1—C10—N3	−46.45 (19)
N1—Mn1—N3—C10	49.29 (13)	O1—C1—C10—N6	11.6 (2)
N2—Mn1—N3—C10	−33.90 (13)	C6—C1—C10—N6	−109.79 (18)
C15—Mn1—N3—C11	−53.69 (18)	C2—C1—C10—N6	138.60 (17)
C16—Mn1—N3—C11	38.68 (18)	C10—N3—C11—C12	1.7 (2)
N1—Mn1—N3—C11	−147.62 (17)	Mn1—N3—C11—C12	−163.23 (14)
N2—Mn1—N3—C11	129.18 (17)	N3—C11—C12—N6	−0.5 (2)
C2—N1—C3—C4	−0.2 (2)	C10—N6—C12—C11	−0.8 (2)
Mn1—N1—C3—C4	169.76 (15)	C13—N6—C12—C11	174.13 (19)
N1—C2—N4—C4	−1.3 (2)	O6—S1—C17—F1	60.3 (3)
C1—C2—N4—C4	−173.59 (17)	O7—S1—C17—F1	−178.0 (2)
N1—C2—N4—C5	174.82 (19)	O5—S1—C17—F1	−57.2 (3)
C1—C2—N4—C5	2.5 (3)	O6—S1—C17—F2	−61.8 (2)
N1—C3—C4—N4	−0.6 (2)	O7—S1—C17—F2	59.9 (2)
C2—N4—C4—C3	1.1 (2)	O5—S1—C17—F2	−179.2 (2)
C5—N4—C4—C3	−175.44 (19)	O6—S1—C17—F3	−178.7 (2)
C7—N2—C6—N5	0.5 (2)	O7—S1—C17—F3	−57.0 (2)
Mn1—N2—C6—N5	179.18 (11)	O5—S1—C17—F3	63.8 (2)

### Hydrogen-bond geometry (Å, º)

**Table:**

*D*—H···*A*	*D*—H	H···*A*	*D*···*A*	*D*—H···*A*
O1—H1···O5	0.72 (2)	1.98 (2)	2.694 (2)	175 (2)

## References

[bb1] Berends, H.-M. & Kurz, P. (2012). *Inorg. Chim. Acta*, **380**, 141–147.

[bb2] Brandenburg, K. (2011). *DIAMOND* Crystal Impact GbR, Bonn, Germany.

[bb3] Breslow, R., Hunt, J. T., Smiley, R. & Tarnowski, T. (1983). *J. Am. Chem. Soc.* **105**, 5337–5342.

[bb4] Brückmann, N. E., Wahl, M., Reiss, G. J., Kohns, M., Wätjen, W. & Kunz, P. C. (2011). *Eur. J. Inorg. Chem.* pp. 4571–4577.

[bb5] Herrick, R. S., Ziegler, C., Jameson, D. & Aquina, C. (2008). *Dalton Trans.* pp. 3605–3609. 10.1039/b802170h 18594710

[bb6] Huber, W., Linder, R., Niesel, J., Schatzschneider, U., Spingler, B. & Kunz, P. C. (2012). *Eur. J. Inorg. Chem.* pp. 3140–3146.

[bb7] Kreiter, C. G., Fiedler, C., Frank, W. & Reiss, G. J. (1995). *J. Organomet. Chem.* **490**, 133–141.

[bb8] Kreiter, C. G., Koch, E.-C., Frank, W. & Reiss, G. J. (1994). *Inorg. Chim. Acta*, **220**, 77–83.

[bb9] Kunz, P. C., Huber, W., Rojas, A., Schatzschneider, U. & Spingler, B. (2009). *Eur. J. Inorg. Chem.* pp. 5358–5366.

[bb10] Niesel, J., Pinto, A., Peindy N’Dongo, H. W., Merz, K., Ott, I., Gust, R. & Schatzschneider, U. (2008). *Chem. Commun.* pp. 1798–1800. 10.1039/b719075a 18379697

[bb11] Oxford Diffraction (2009). *CrysAlis PRO.* Oxford Diffraction Ltd., Oxford, UK.

[bb12] Sheldrick, G. M. (2008). *Acta Cryst.* A**64**, 112–122. 10.1107/S0108767307043930 18156677

[bb13] Stamatatos, T. C., Efthymiou, C. G., Stoumpos, C. C. & Perlepes, S. P. (2009). *Eur. J. Inorg. Chem.* pp. 3361–3391.

[bb14] Tang, C. C., Davalian, D., Huang, P. & Breslow, R. (1978). *J. Am. Chem. Soc.* **100**, 3918–3922.

[bb15] Westrip, S. P. (2010). *J. Appl. Cryst.* **43**, 920–925.

